# *Salmonella enterica* Serovar Infantis in Broiler Chickens: A Systematic Review and Meta-Analysis

**DOI:** 10.3390/ani14233453

**Published:** 2024-11-28

**Authors:** Alexandros Georganas, Giulia Graziosi, Elena Catelli, Caterina Lupini

**Affiliations:** Department of Veterinary Medical Sciences, University of Bologna, 40064 Ozzano dell’Emilia, BO, Italy; alexandros.georganas@studio.unibo.it (A.G.); giulia.graziosi2@unibo.it (G.G.); caterina.lupini@unibo.it (C.L.)

**Keywords:** *Salmonella* Infantis, broiler, chicken, systematic review, meta-analysis, prevalence

## Abstract

*Salmonella enterica* subsp. *enterica* serovar Infantis poses a growing threat to public health, due to its increasing prevalence worldwide and its association with high levels of antimicrobial resistance. Among livestock, *S.* Infantis is especially isolated from broilers. Following the Preferred Reporting Items for Systematic Reviews and Meta-Analyses (PRISMA) guidelines, a systematic review was conducted by searching in three databases (Web of Science, Scopus, and PubMed) for English-language studies (1957–2023) that reported the prevalence of *S.* Infantis in broiler farms. Eligible studies included epidemiological investigations conducted in broiler chickens by sampling the house environment (flock-level prevalence) or the birds (individual-level prevalence). A random-effect model was applied to calculate *S.* Infantis pooled prevalence estimates with 95% confidence intervals (CIs). Furthermore, to assess between-study heterogeneity, the inconsistency index statistic (I^2^) was calculated. Among 537 studies retrieved, a total of 9 studies reporting flock-level prevalence of *S.* Infantis and 4 reporting individual-level prevalence were retained for analysis. The flock-level pooled prevalence was estimated to be 9% (95% CI: 1–26%) and a high between-study heterogeneity was found (I^2^ = 99%, *p* < 0.01). Concerning individual-level prevalence, a meta-analysis was not performed due to the scarcity of eligible studies. The data presented underscore the significant occurrence of *S.* Infantis in broilers at the farm level. By summarizing the existing literature, this work provides useful insights for conducting future surveys of *Salmonella* spp. in live broiler chickens as a preliminary step for developing more efficient control strategies.

## Simple Summary

*Salmonella enterica* subsp. *enterica* serovar Infantis is an emergent and zoonotic pathogen whose prevalence in humans has increased globally in recent decades. Among livestock, broiler chickens are considered as a major source of *S.* Infantis in many regions, including the United States, Europe, and Latin America. This *Salmonella* serovar is also strongly associated with high levels of antimicrobial and multidrug resistances. By conducting a systematic review and meta-analysis, this study summarizes data on *S.* Infantis occurrence in broilers sampled at the farm level. The results obtained underscore the high infection burden of *S.* Infantis in broiler chickens.

## 1. Introduction

Salmonellosis is among the major causes of foodborne gastrointestinal infection in humans worldwide [1] and the second cause following campylobacteriosis in the European Union (EU) [2]. Particularly, non-typhoidal *Salmonella* spp. (NTS) are one of the bacterial pathogens with the highest disease burden among human foodborne enteric diseases [3]. Poultry products are considered as a major source of human infection in cases of foodborne salmonellosis caused by NTS [4]. In the US and Japan, approximately 70% of human cases of foodborne salmonellosis were attributable to consumption of contaminated chicken or turkey meat and egg products [5,6,7,8]. Among NTS, *Salmonella enterica* serovar Infantis was the fourth main cause of human salmonellosis in the EU in 2022 [9] and the sixth most common *Salmonella* serotype in the US in 2019 [10], with an outbreak of a persistent multidrug-resistant (MDR) *S.* Infantis strain, which occurred in 2018, being linked to raw chicken products [11]. For Europe, since 2014, *S.* Infantis has become the no. 1 serovar isolated in broilers and their meat [3], thus representing a potential route for human illness [3,12,13]. However, in the analysis of Powell and Williams [14], it was shown that there was a 4 yr lag between the increase in the incidence of human illness due to *S.* Infantis which started in 2011 and the increase in chicken carcass prevalence which started in 2015, suggesting that one or more other transmission route of human illness likely contributed to the increase in incidence of human illness due to *S.* Infantis in the United States (US). These transmission routes, other than consumption of chicken products, include non-chicken foodborne, waterborne, person-to-person, animal contact, and environmental routes [14]. Moreover, it has also been suggested that the strains containing the new megaplasmid termed ‘plasmid of emerging *S*. Infantis’ (pESI) that circulate in the poultry industry may have a selective disadvantage for causing infection in humans, thus resulting in less frequent observation of those strains [15].

The various traits of the emerging *S.* Infantis strains carrying the pESI megaplasmid, such as their persistence throughout the food chain, resistance to disinfectants, elevated tolerance to environmental mercury, augmented abilities to form biofilms, higher tolerance to thermal, acid, and osmotic stress, and, most importantly, the widespread presence of antimicrobial or MDR clones, pose challenges to mitigate these strains both in poultry farming and agriculture [3,16]. Following its first report in Israel [17], the megaplasmid pESI which harbors *bla*_CTX-M-65_ and other antibiotic-resistant genes has been found in *S.* Infantis isolates from chicken, food, and human clinical cases in South America, North America, Europe, Africa, and Asia [18,19]. The *bla*_CTX-M_ genes encode CTX-Ms, which are the most dominant extended-spectrum β-lactamase enzyme types that incapacitate β-lactam-type antibiotics [20]. A recent analysis of whole genome sequences of *S.* Infantis from 74 countries found that 73% of poultry isolates were MDR, with 71% carrying the pESI megaplasmid, compared to 35% of human isolates, with 32% carrying the pESI megaplasmid [15]. Evidence in a mouse model showed that after infection with *S.* Infantis, pESI can be horizontally transferred to the gut microbiota [21], which could result in the spread of multidrug resistance (resistance to ≥3 antimicrobial classes) and elevated virulence to other pathogens [22]. In addition to the increased frequency of isolation of MDR *S.* Infantis from human infections over the last decade [23], this evidence raises questions about its implications for public health, especially when the pESI megaplasmid is present; however, these remain unclear as the repercussions of infections in humans, such as the severity of infection and the implication of antimicrobial resistance, have yet to be explored [22]. Nevertheless, the pESI plasmid has been shown to be a major driver for antimicrobial resistance in the global *S.* Infantis population and high levels of multidrug resistance, with the highest level at 81% in South America, have been observed in many regions, thus suggesting a major threat to public health [15].

A large proportion of the global population consumes chicken meat and eggs to partially meet their protein needs [24]. Furthermore, demand for animal products is projected to increase and, to meet consumer demand, world meat production is forecasted to double at an amount of 455 million tons by 2050 [25]. In line with the above, globalization and a rise in international trade of foodstuffs such as poultry meat have contributed to the dispersion of new *Salmonella* serovars [26]. It is also probable that extreme weather events and climate change may increase the spread of *Salmonella* spp. due to the increased temperatures, which are favorable for their growth [27]. Moreover, extreme heat due to climate change may induce heat stress in poultry, a predisposing factor to infection by bacteria including *Salmonella* spp. [27]. Inappropriate adoption of good production practices, such as keeping a higher than recommended stocking density, can stress the poultry flocks, resulting in an elevated occurrence, persistence and spread of NTS [27,28,29]. The same applies to interventions such as thinning or partial depopulation, which is common practice in some production systems [30]. Thinning in conjunction to poor biosecurity practices, such as substandard cleaning and disinfection of protective personal equipment or crates when visiting more than one farm, can increase the risk of *Salmonella* spp. colonization due to passive transfer. Furthermore, thinning constitutes a stressor for birds due to feed withdrawal and distress during catching, leading to increased vulnerability to disease [31]. Altogether, these factors pose new challenges to salmonellosis control, and innovative intervention strategies are necessary to control *S.* Infantis in poultry flocks.

Due to the surge of *S.* Infantis serovar in broilers and the burden of chicken-origin *S.* Infantis infections in humans, the present work aims to summarize the evidence on *S.* Infantis occurrence in broiler chickens in farms on a global level by conducting a systematic review of the literature and meta-analysis of prevalence data. Data on the antimicrobial resistance of the *S.* Infantis strains isolated in the eligible studies are summarized. By doing so, the research is expected to tentatively give directions for future studies focusing on the epidemiology of *S.* Infantis at the farm level. 

## 2. Materials and Methods

### 2.1. Study Design and Systematic Review Protocol

The Preferred Reporting Items for Systematic Reviews and Meta-Analyses Protocols (PRISMA-P) and the PRISMA 2020 Statement were used to search and identify the references [32] (Supplementary Material Table S1). The systematic review was registered in the International Prospective Register of Systematic Reviews (PROSPERO) under the number CRD42024533765. A literature search was conducted from 1 October 2023 to 10 December 2023. Three databases were accessed, namely PubMed (https://pubmed.ncbi.nlm.nih.gov), Web of Science (https://apps.webofknowledge.com/), and Scopus (https://www.scopus.com/). The full search strategies including the search strings used in each database and the number of articles retrieved are included in Table 1. In the three databases queried, the Boolean operators ‘OR’ and ‘AND’ were used, and relevant MeSH terms were added in PubMed. Filters such as publication date, article type (e.g., research articles, reviews), or language were not applied in the literature search. The reference lists of eligible studies were also screened to find other relevant contributions.

### 2.2. Literature Research Strategy

The tool Rayyan was used [33] to identify duplicates and screen the articles. Titles and abstracts were screened by two independent investigators (A.G. and G.G.) based on predetermined inclusion and exclusion criteria (Supplementary Material Table S1). The articles were selected for full-text review if they investigated *Salmonella* spp. in broiler chickens, if they were in English and if they reported original data. Finally, the full text of the articles was downloaded and independently assessed for eligibility, data analysis and extraction by A.G. and G.G. In case of potential disagreements, a third experienced author in the avian pathology field (C.L.) was consulted. The articles would pass the screening after full-text review if the following criteria were met: (1) the full English text could be retrieved; (2) the scientific articles reported original data (no duplicated data); (3) the article reported isolation or detection of *S.* Infantis regardless of laboratory methods used; (4) samples were collected from individual broiler chickens (e.g., cloacal swabs, internal organs such as liver and spleen) or from broiler chicken houses (e.g., litter samples, dust, fecal or cecal droppings); (5) the prevalence of infection could be calculated given the available information found in the article; and (6) the country and location where the study took place were reported. The two outcomes of interest were individual-level prevalence from testing single birds and flock-level prevalence from testing samples collected in chicken houses. For flock-level prevalence, a flock positive to *S.* Infantis was considered positive if at least one sample was positive. In the present work, the following definition of flock is used: “*A flock is defined as a group of chickens (…), belonging to the same herd, with the same sanitary and immune status, reared in the same room or barn, and having the following common characteristics: species, category (breeders, production), type (laying, broiler), stage of production (age), sanitary status”* [34]. Studies were excluded if the samples originated from birds with suspected clinical salmonellosis and if fewer than 20 samples were collected. 

### 2.3. Data Extraction and Management

From each eligible article, the following information was extracted and included in a data extraction sheet (Microsoft Excel 2016, version 2402), where available: reference ID (numbering each included reference), first author, year of publication, country, sampling period, sample type, total number of samples tested, total number of samples positive for *S.* Infantis, detection method(s), resistance of *S.* Infantis to antimicrobials, and methods for antimicrobial resistance diagnostics. Studies were also grouped into continents where the samples were collected to facilitate data analysis and reporting. If prevalence data of *S.* Infantis were reported as percentage, raw numbers were calculated by transforming the percentage to the closest integer, considering the total number of samples collected.

### 2.4. Study Risk of Bias and Quality Assessment

The quality assessment of eligible studies was conducted independently by A.G. and G.G. applying the Joanna Briggs Institute critical appraisal checklist for prevalence studies (https://jbi.global/critical-appraisal-tools (accessed on 26 November 2024)). The dataset presented in the current study was suitable for the assessment of the Joanna Briggs Institute tool, even though the checklist is intended for human studies [35].

### 2.5. Statistical Analysis

Extracted data from the eligible articles were analyzed using the R software (version 4.3.2., http://cran.r-project.org/) using the ‘meta’ package. Prevalence of *S.* Infantis in broiler chickens and its 95% confidence interval (CI) were calculated using a random-effects model following the double-arcsine transformation of data [36]. The description of the heterogeneity (or variability) is essential in a meta-analysis since the sampling methods and experimental methodologies of the primary studies assessed were not identical [37]. Therefore, to estimate the between-study heterogeneity, Cochran’s Q and the inconsistency index (I^2^) were used on the pooled estimates. The interpretation of the I^2^ statistic was as follows: small (<25%); medium (25–50%); and large (>75%) [38].

## 3. Results

### 3.1. Literature Searches

**Figure 1:**
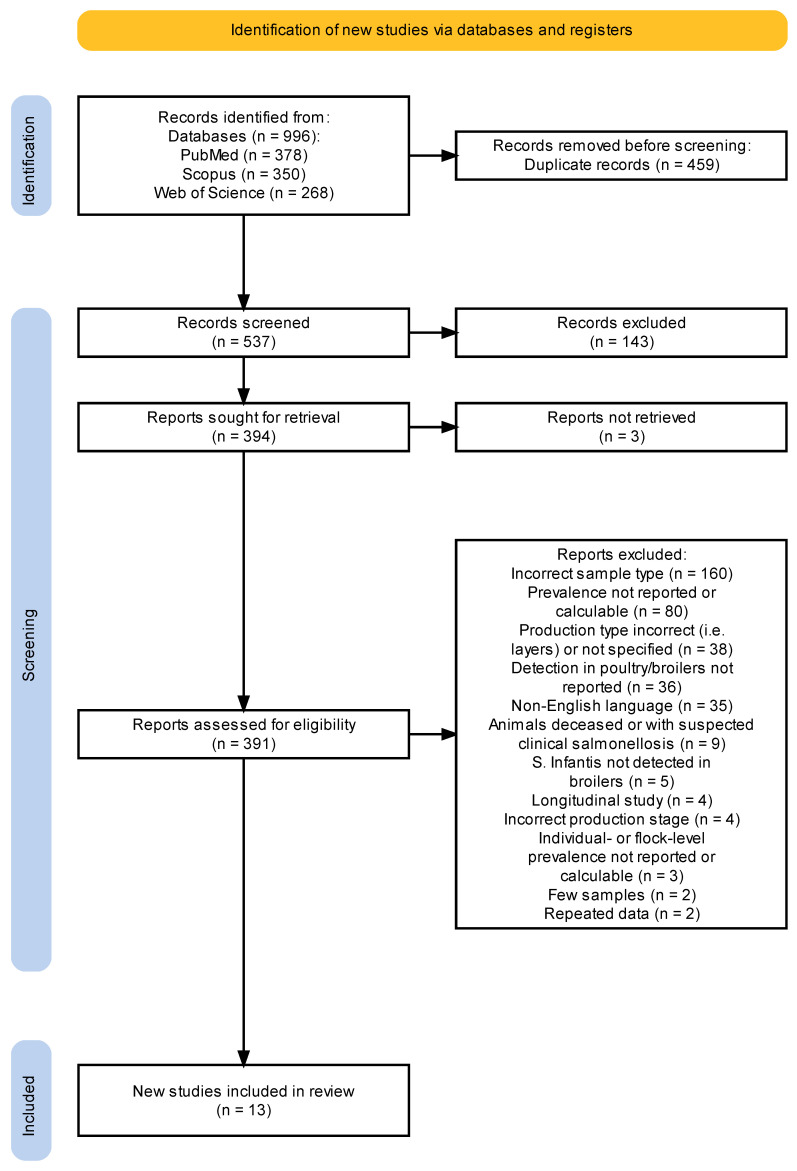
PRISMA flowchart depicting identification, screening, and selection of eligible studies assessed in the systematic review, 1957–2023.

The PRISMA flowchart on the selection of the eligible studies is depicted in Figure 1. Following removal of the duplicate studies retrieved from the search of three databases (Table 1), 537 articles were assessed for title and abstract screening. Of these articles, 394 (73.4%) were deemed potentially relevant and the full text of which was sought for retrieval. Of those, the full text of 391 articles was available and was therefore assessed. Finally, 13 articles were considered eligible for inclusion following full-text review, which corresponded to 2.4% (13/537 articles) of the retrieved articles excluding the duplicate studies. Of the fourteen eligible studies, nine studies reported flock-level prevalence of *S.* Infantis in broiler chickens (Table 2) and four studies individual-level prevalence (Table 3). Due to the limited number of the latter studies, a meta-analysis was conducted only on the flock-level prevalence.

With respect to the eligible studies reporting flock-level prevalence of *S.* Infantis, most of these were conducted in Europe (n = 4), followed by North America (n = 2), South America (n = 1), Africa (n = 1), and Asia (n = 1). In total, 72,602 flocks of broiler chickens were tested (Table 2), of which 600 flocks were positive for *S.* Infantis. With respect to the type of sample submitted for microbiological investigation, the majority of the studies (6/9, 66.6%) analyzed litter samples, while the remaining three studies tested fecal or cecal droppings. In four/nine (44.4%) studies reporting flock prevalence, *S.* Infantis was the predominant serotype [39,40,41,42]. In the study of Long et al. [42], *S.* Infantis and *S.* Heidelberg were the most prevalent serotypes isolated from the same number of flocks (n. 16), but more isolates of the latter serotype were found (144 versus 66). In three/nine (33.3%) eligible studies, *S.* Infantis was the second dominant serotype [22,43,44]. In the study of El-Hage et al. [44], *S.* Infantis was the second most prevalent serotype, after *S*. Enteritidis. In two/nine (22.2%) eligible studies, carried out in Austria in the period 2005–2006 and in Poland in 2014–2016, *S.* Infantis was the fourth most dominant serovar isolated [45,46].

For individual-level prevalence, studies were concentrated in Asia (n = 3) and South America (n = 1). No studies reporting prevalence of *S.* Infantis in broiler chickens in Europe, North America and Africa were eligible from our criteria. Regarding the type of sample submitted for microbiological investigation, two/four (50%) analyzed feces collected from individual birds and two/four (50%) analyzed cloacal swabs. In the four eligible studies, *S.* Infantis was the predominant serotype [47,48,49,50].

**Table 2:** Summary of eligible observational studies reporting flock prevalence of *Salmonella* Infantis in broiler chickens included in the meta-analysis.

Reference.	Continent/Location	Sampling Period	Sample Type	Samples Per Flock	Detection and Identification Method	Total No. of Flocks Sampled	No. of *S.* Infantis Positive Flocks (%)
	Europe						
Mughini-Gras et al. [22]	The Netherlands	July 2018 to May 2019	Fecal droppings	3 per flock, 12 fresh droppings per sample	(i) Isolation (enrichment) (ii) Molecular serotyping (xMAP *Salmonella* serotyping assay, Luminex, MALDI-TOF for confirmation of genus *Salmonella*) (iii) Sequencing (in silico multi-locus sequence typing)	379	14 (3.7%)
Cargnel et al. [39]	Belgium	2012 to 2018	Litter	1	(i) Isolation (enrichment, ISO 6579:2017) [51] (ii) Serotyping	66,706	342 (0.5%)
Witkowska et al. [46]	Poland	2014 to 2016	Fecal droppings	NR ^a^	(i) Isolation (enrichment) (ii) Biochemical tests (PN-EN ISO-6579:2003 [52], item 9.5.3) (iii) Serotyping (slide agglutination; PN-EN ISO-6579:2003, item 9.5.4)	4331	13 (0.3%)
Lassnig et al. [45]	Austria	October 2005 to September 2006	Litter	5	(i) Isolation (enrichment, ISO 6579:2002 [53] method) (ii) Biochemical tests (iii) Serotyping	363	2 (0.6%)
	South America						
Burnett et al. [40]	Galapagos Islands	February 2016 to April 2017	Litter	1	(i) Isolation (enrichment, ISO 6579-1:2007) [54] (ii) Serotyping	22	5 (22.7%)
	Asia						
El Hage et al. [44]	Lebanon	October 2014 to October 2015	Litter	1	(i) Isolation (enrichment, NF EN ISO 6579:2002) (ii) Biochemical tests (NF EN ISO 6579:2002) (iii) Serotyping (slide agglutination, NF EN ISO 6579:2002) (iv) Pulsed-field gel electrophoresis	159	11 (6.9%)
Sasaki et al. [41]	Japan	November 2007 to February 2010	Cecal droppings	5	(i) Isolation (enrichment) (ii) Biochemical tests (iii) Serotyping (slide and tube agglutination)	288	176 (61.1%)
	North America						
Poppe et al. [43]	Canada	December 1989 to May 1990	Litter	12	(i) Isolation (enrichment) (ii) Biochemical tests (iii) Serotyping	294	21 (7.1%)
Long et al. [42]	Canada	June 1978 to January 1979	Litter	15	(i) Isolation (enrichment) (ii) Biochemical tests (iii) Serotyping (slide agglutination)	60	16 (26.7%)

^a^ NR = not reported.

**Table 3:** Summary of eligible observational studies reporting individual-level prevalence of *Salmonella* Infantis in broiler chickens.

Reference	Continent/Location	Sampling Period	Sample Type	Detection and Identification Method	Total No. of Broiler Chickens Sampled	No. of *Salmonella* spp. Positive Samples	No. of *S.* Infantis Positive Samples
	Asia						
Badouei et al. [48]	Iran	September to October 2013	Feces	(i) Isolation (enrichment) (ii) Biochemical tests (iii) Serotyping (slide agglutination) (iv) Molecular detection (multiplex and/or two-step nested polymerase chain reaction, PCR) for *Salmonella* serovar confirmation	153	30 (19.6%)	23 (15.0%)
Cui et al. [50]	China	August to September 2013	Cloacal swabs	(i) Isolation (enrichment) (ii) Molecular detection (PCR) for *Salmonella* spp. confirmation (iii) Serotyping (slide agglutination)	640	56 (8.8%)	17 (2.7%)
Ishihara et al. [49]	Japan	1999	Feces	(i) Isolation (first and delayed secondary enrichment cultures) (ii) Biochemical tests (iii) Serotyping (slide and tube agglutination)	155	56 (36.1%)	35 (22.6%)
	South America						
Khan et al. [47]	Trinidad and Tobago	February to July 2019	Cloacal swabs	(i) Isolation (enrichment) (ii) Biochemical tests (iii) Serotyping (slide agglutination test)	675	15 (2.2%)	11 (1.6%)

### 3.2. Antimicrobial Resistance Genes in S. Infantis Isolates

Antimicrobial resistance and multidrug resistance of *S.* Infantis isolates were reported in most of the eligible studies (8/13 studies), and the diagnostic methods used are reported in Table 4. In the study of Mughini-Gras et al. [22], all but one isolate carried a pESI mega-plasmid and all isolates belonged to the same sequence type (ST) 32. High prevalence of five antibiotic resistance genes encoding resistance against four different classes of antibiotics, namely aminoglycosides (*aadA1* and *aph* (3′)-Ic), sulphonamide (*sul1*), tetracycline (*tetA*), and trimethoprim (*drfA14*), was found. Burnett et al. [40] reported MDR *S.* Infantis in Galapagos; the isolates belonged to ST32 and were closely related, following phylogenetic characterization, to *S.* Infantis isolated in the US and South America. Furthermore, all *S.* Infantis isolates possessed mutation D87Y in the *gyrA* gene conferring decreased susceptibility to ciprofloxacin. *S.* Infantis isolates possessing tetracycline (*tetA*), trimethoprim (*dfrA14*), and sulfonamide (*sul1*) resistance genes displayed the corresponding resistance phenotypes. Similarly, multiple aminoglycoside resistance genes, namely *aph(4)-la*, *aadA1*, *aac(3)-IVa*, and *aph(30)-Ia*, were detected in the *S.* Infantis genomes and corresponded to gentamicin-resistant phenotypes. *S.* Infantis harbored the ESBL-producing *bla*_CTX-M-65_ gene in an IncFIB-like plasmid and was characterized by reduced fluoroquinolone susceptibility [40]. In the study of El Hage et al. [44], *S.* Infantis isolates showed high phenotypic resistance to tetracycline (99%) and streptomycin (88.2%), and, to a lesser extent, to trimethoprim (2.4%) and trimethoprim–sulfamethoxazole. Similarly, high antimicrobial phenotypic resistance rates (>90% of isolates) against oxytetracycline and dihydrostreptomycin were observed among *S.* Infantis isolates in the study of Sasaki et al. [41]. Antimicrobial resistance was also found against ampicillin, trimethoprim, kanamycin, cefazolin and ceftiofur, while fewer isolates exerted resistance against nalidixic acid, bicozamycin and chloramphenicol [41]. In the study of Badouei et al. [48], most *S.* Infantis isolates exerted phenotypic multidrug resistance against amoxicillin/clavulanic acid, colistin, tetracyclines, nalidixic acid, furalozidone, lincospectin and nitrofurantoin, and, to a lesser extent, against aminoglycosides anamycin, neomycin and streptomycin, chloramphenicol, florfenicol, flumequine, ciprofloxacin, and cefazolin, with 12 resistance patterns. In the study of Cui et al. [50], resistance against nalidixic acid, ampicillin, doxycycline, cefazolin, gentamicin, chloramphenicol and trimethoprim/sulfamethoxazole was observed with six resistance patterns. Likewise, in the study of Ishihara et al. [49], high resistance rates (61.3%) to dihydrostreptomycin, kanamycin, oxytetracycline and trimethoprim were found in the *S.* Infantis isolates. In the study of Khan et al. [47], most isolates (85.7%) were MDR, and seven resistance patterns were found, with doxycycline–ceftriaxone–gentamicin–kanamycin–sulfamethoxazole being the predominant pattern. All (100.0%) isolates of *S.* Infantis were also resistant to doxycycline [45]. In the study of Long et al. [42], all *S.* Infantis isolates were susceptible to all antimicrobials tested (Table 4). Antimicrobial resistance was not investigated in the studies of Cargnel et al. [39], Witkowska et al. [46] and Poppe et al. [43], whereas in the study of Lassnig et al. [45], the investigation was not serovar-specific.

### 3.3. Quality Assessment

According to the quality assessment conducted on the Joanna Briggs Institute critical appraisal checklist for prevalence studies, all the contributions met the required standard.

### 3.4. Statistical Analysis

**Figure 2:**
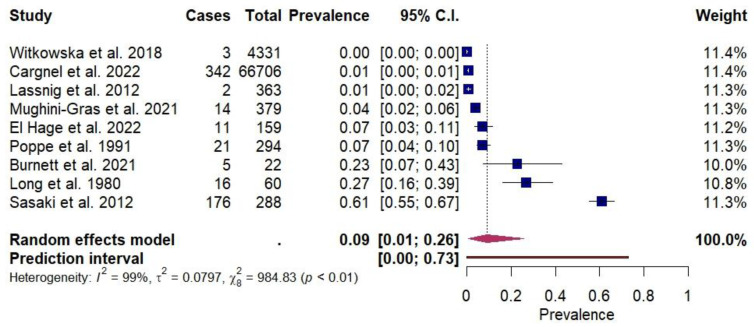
Forest plot of the random-effects meta-analysis of flock-level prevalence of *Salmonella enterica* Infantis. *I*^2^ (inverse variance index), *τ*^2^ = the between-study variance, *χ*^2^ and *p*-value of the Cochran’s Q test for heterogeneity [6,22,39,40,41,42,43,44,45].

The pooled flock-level prevalence of *S.* Infantis in broilers was 9% (95% CI: 1–26%, I^2^ = 99%) (Figure 2). Due to the limited number of studies retrieved from the literature, subgroup analyses based on geographic area or sample type were not conducted.

## 4. Discussion

### 4.1. Summary of Evidence

To the best of our knowledge, this is the first meta-analysis on the prevalence of *S.* Infantis in broiler chickens sampled at the farm level. Despite the limited data retrieved from the literature, the present work underscores the relevance of *S.* Infantis in broiler farms; however, due to the wide 95% CI (1–26%), the pooled flock-level prevalence (9%) obtained in the meta-analysis should be interpreted with caution. Indeed, given the high degree of between-study heterogeneity, the pooled estimate needs to be considered together with its 95% CI.

Various reports have indicated the widespread surge of *S.* Infantis in the poultry sector, especially in broilers, thus possibly being a route for human infections [2,3,15]. From the literature search hereby conducted, a total of 13 articles concerning *S.* Infantis detection at the broiler farm level were deemed eligible. To interpret the low number of studies included, it is important to consider that the purpose of most of the studies investigating *Salmonella* spp. in broiler chickens was the analysis of antimicrobial resistance and seroprevalence of *S.* Infantis at the farm level, and many studies involved sampling activities performed at slaughterhouses or on retail meat, which have not been considered in the present study (Figure 1).

For the EU, only four studies were deemed eligible for the systematic review hereby presented, and *S.* Infantis was found to be the first to the fourth most frequently isolated *Salmonella* serovar in flocks [22,39,45,46]. With respect to surveillance data from Member States, *S*. Infantis was the main serovar isolated from broilers in 2022 [9]. Moreover, studies reporting data on broiler carcasses and meat performed in Italy, Belgium, Romania and The Netherlands found *S.* Infantis as the most common *Salmonella* serotype [22,55,56,57,58,59]. 

With respect to Asia, a total of three studies, two from Japan and one from Lebanon, were deemed eligible [41,44,49]. In Japan, the high flock and individual-level prevalence found in the two Japanese studies included in this review [41,49] is in line with results reporting *S.* Infantis as the predominant serovar isolated within a timeframe from 1998 to 2010 in broiler chickens sampled at the abattoir or in raw chicken parts [60,61]. More recently, prevalence of *S.* Infantis in cecal samples of broiler chickens collected at a poultry processing plant was found at approximately 5% between 2009 and 2016 [62,63], with increased resistance against extended-spectrum cephalosporins mediated by extended-spectrum β-lactamases and AmpC β-lactamases [64]. In China, a study hereby included found that *S.* Infantis was the second most dominant serotype in adult broiler chickens [50], whereas heterogeneous results were reported at the slaughterhouse level, with other *Salmonella* serovars being more frequently isolated [65,66]. With respect to other countries in which no studies on *S.* Infantis prevalence at the farm level were available, this serovar was predominant in chicken carcasses from poultry processing plants as reported for Iran and South Korea [48,67,68,69].

Concerning Central and South America countries, namely Brazil, Ecuador, Colombia, Trinidad and Tobago and the Galapagos Islands, the predominance of *S.* Infantis serovar among *Salmonella* spp. is documented for both broilers either sampled at the farm level and at slaughterhouses [40,70,71,72,73,74,75]. High resistance rates to antibiotics of *S.* Infantis and isolation of MDR strains are frequently reported in broiler batches at abattoirs or in broiler litter samples [40,72,74,75]. 

With respect to North America, the only two studies deemed eligible confirmed the high frequency of *S.* Infantis isolation from litter samples in Canadian broiler houses [42,43]. All the studies retrieved for the US included sample types not eligible for the systematic review hereby proposed (e.g., broiler carcasses) or did not include any data on individual- or flock-level prevalence of the *S.* Infantis serovar. Data from US processing plants on the overall *Salmonella* spp. incidence in broiler carcasses showed a decrease from 9 to 6.57% between 2016 and 2020 [76]. However, the rate of isolation of *S.* Infantis from raw poultry meat samples in the US, around the same time frame, has increased from 4–10% to over 30%, which is probably attributable to the presence of the pESI plasmid that can confer an increased resistance to *Salmonella* spp. isolated in intensive-farming environments [19,77,78]. Regional differences in Salmonella spp. prevalence in the US include higher proportions of *S.* Infantis and *S.* Typhimurium in broiler carcasses and intact parts from poultry processing facilities in the Atlantic region and higher proportions of serovar *S.* Schwarzengrund in the southeast [76]. One hypothesis of the emergence of *S.* Infantis in broilers is that climate or environmental conditions in the Atlantic may have promoted the presence of *S.* Infantis or suppressed other serotypes such as *S.* Kentucky. Another prevailing hypothesis is that the eradication of other serotypes such as *S.* Kentucky [76] or *S.* Enteritidis and *S.* Typhimurium, due to effective implementation of control programs such as vaccination of breeders and laying hens against these serovars, likely established an ecological niche favorable for the proliferation and spread of *S.* Infantis [16,79].

With respect to Africa, no studies reporting prevalence of *S.* Infantis in broiler chickens were eligible from our criteria. However, according to a systematic review and metanalysis of Thomas et al. [8], the most prevalent *Salmonella* serovars circulating in African poultry up to 2016 are *S.* Enteritidis (20.8%), *S.* Typhimurium (13.9%), and *S.* Typhi (7.8%).

Several risk factors can be identified for the introduction and persistence of *S.* Infantis in broiler farms. In a study conducted in The Netherlands, Mughini-Gras et al. [22] found that *S.* Infantis was isolated more often in flocks in which salinomycin was used and in flocks in which thinning was applied or litter quality was poor. It was also reported that employing external cleaning companies, incorporating wheat in diets, and vaccination against infectious bronchitis resulted in lower prevalence of *S.* Infantis [22]. Furthermore, whole genome sequencing analysis suggested that the introduction of MDR *S.* Infantis in different premises may be able to be spread by the movement of equipment or personnel [80]. Appropriate and strict biosecurity measures are the first line of defense, such as constructing the farm at a proper geographical location; appropriate design of the buildings and positioning of equipment; and well-developed operational protocols with a focus on potential sources of infection and the flow of people, materials, feed, eggs and flocks to and from the farm [31]. In the EU, inactivated vaccines for breeders and layers against *S.* Infantis are available commercially and recombinant vaccines are being developed [16]. In recent years, innovative strategies have also been developed to control *S.* Infantis, such as the use of bacteriophages as biosanitizers against persistent *S.* Infantis strains [16,81]; however, a limited number of studies have been conducted on the use of bacteriophages at the farm level, and concerns are expressed on such use, including the unknown effectiveness and potential genotoxicity [27]. However, it is argued that many prevention and control measures of *Salmonella* spp. in poultry feed cannot eliminate the pathogen due to the continual possibility of recontamination [82]. *S.* Infantis has been isolated from biscuit meal, minerals, wheat and soya [83]. Disinfecting feeder lines and external areas in addition to the broiler houses is essential to eliminate *S.* Infantis [80]. It is crucial to consider risk factors to combat *Salmonella* spp., given that *Salmonella* spp. contamination, including *S.* Infantis, which takes place in the early stages of the broiler supply chain, can be transmitted along the food chain [84,85]. In fact, inadequately cleaned and disinfected transport crates contaminated with *Salmonella* spp. may lead to cross-contamination at the slaughterhouse and back to the farm level [59]. Furthermore, insects such as *Typhaea stercorea* (Coleoptera: Mycetophagidae) can be carriers of *S.* Infantis in broiler houses [86]. 

Various factors can influence the survival and/or distribution of *Salmonella* spp. in broiler houses and need to be taken into consideration when designing surveillance or monitoring strategies. These include the season, weather (low rainfall and moderate temperatures), and the type of housing (cage or floor). Also, the sample type to be tested should be carefully chosen, since this could influence the results obtained. For this reason, a multi sample-type approach has high positive predictive value, even when the diagnostic test sensitivity and the environmental prevalence are low [87]. Further, completeness of data reported is encouraged for a comprehensive interpretation of results and for facilitating the direct comparability of the results between the studies. This includes reporting baseline characteristics of the flock (e.g., age, production type and stage) and, with respect to the microbiological investigations, the prevalence of each *Salmonella* serotype.

### 4.2. Limitations

Among the constraints identified regarding the present work, the scarcity of eligible studies that met our predetermined inclusion and exclusion criteria on the prevalence of *S.* Infantis in individual live broiler chickens sampled at the farm level was recognized as a limitation of the current systematic review and meta-analysis. Hence, the true geographic distribution and prevalence of *S.* Infantis in live broilers cannot be interpreted. We also suggest the existence of possible research which may not have been accessible through the search strategy adopted. 

With respect to samples collected at the farm level, the weight of litter or fecal droppings was not always stated in the methods by the respective authors. Indeed, it has been shown that the weight of fecal samples might affect the prevalence estimates [88]. Furthermore, the ISO 6579 method for the detection of *Salmonella* spp. was employed in six/nine eligible studies included in the meta-analysis. Thus, some variation in the prevalence estimates may be explained by the different methods used among the studies. Regarding the test sensitivity and specificity of the methods, they were not corrected for the prevalence estimates; thus, the apparent prevalence may differ from the true prevalence and may be affected by the sampling and testing methodology [8]. In addition, as shown in Table 4, even though the methods to test antimicrobial resistance were consistent, there was variation in the antimicrobials tested and the guidelines used to interpret the results, that is, guidelines mainly from the European Committee on Antimicrobial Susceptibility Testing or the Clinical and Laboratory Standards Institute. Lastly, quantification of publication bias via statistical tests was not conducted due to the absence of specific tools applicable to studies on proportions [35,89,90]. 

## 5. Conclusions

Considering the emerging challenge of *S.* Infantis in the broiler sector and its relevance as a zoonotic bacterium, we considered it of high importance to summarize the up-to-date knowledge on its prevalence in broiler chickens at the farm level. By gathering the evidence from the literature, the results presented hereby could help to design future epidemiological surveys to detect *S.* Infantis in poultry, as a first step to enhance evidence-based control strategies of this serovar in the broiler industry. In this respect, consistent monitoring of *S.* Infantis and the potential antimicrobial resistance present, careful application of biosecurity measures, and the development and use of a universal vaccine against *S.* Infantis are essential to reduce its presence in broiler production. 

## Figures and Tables

**Table 1:** Search strategies for the literature research of studies on *Salmonella* Infantis in broilers retrieved from the PubMed, Scopus, and Web of Science databases (1957–2023).

Database	Number of Articles Retrieved	Search String/Terms and Limits
PubMed	378	(((“salmonella”[MeSH Terms] OR “salmonella”[All Fields] OR “salmonellas”[All Fields] OR “salmonella s”[All Fields] OR “salmonellae”[All Fields]) AND “Infantis”[All Fields]) OR (“s”[All Fields] AND “Infantis”[All Fields])) AND (“chicken s”[All Fields] OR “chickens”[MeSH Terms] OR “chickens”[All Fields] OR “chicken”[All Fields] OR “gallus”[All Fields] OR (“broiler”[All Fields] OR “broiler s”[All Fields] OR “broilers”[All Fields]) OR (“birds”[MeSH Terms] OR “birds”[All Fields] OR “bird”[All Fields])) AND (“epidemiology”[MeSH Subheading] OR “epidemiology”[All Fields] OR “incidence”[All Fields] OR “incidence”[MeSH Terms] OR “incidences”[All Fields] OR “incident”[All Fields] OR “incidents”[All Fields] OR (“epidemiology”[MeSH Subheading] OR “epidemiology”[All Fields] OR “prevalence”[All Fields] OR “prevalence”[MeSH Terms] OR “prevalance”[All Fields] OR “prevalences”[All Fields] OR “prevalence s”[All Fields] OR “prevalent”[All Fields] OR “prevalently”[All Fields] OR “prevalents”[All Fields]) OR (“isolate”[All Fields] OR “isolate s”[All Fields] OR “isolated”[All Fields] OR “isolates”[All Fields] OR “isolating”[All Fields] OR “isolation and purification”[MeSH Subheading] OR (“isolation”[All Fields] AND “purification”[All Fields]) OR “isolation and purification”[All Fields] OR “isolation”[All Fields] OR “isolations”[All Fields]) OR (“survey s”[All Fields] OR “surveyed”[All Fields] OR “surveying”[All Fields] OR “surveys and questionnaires”[MeSH Terms] OR (“surveys”[All Fields] AND “questionnaires”[All Fields]) OR “surveys and questionnaires”[All Fields] OR “survey”[All Fields] OR “surveys”[All Fields]) OR (“detect”[All Fields] OR “detectabilities”[All Fields] OR “detectability”[All Fields] OR “detectable”[All Fields] OR “detectables”[All Fields] OR “detectably”[All Fields] OR “detected”[All Fields] OR “detectible”[All Fields] OR “detecting”[All Fields] OR “detection”[All Fields] OR “detections”[All Fields] OR “detects”[All Fields]) OR (“occur”[All Fields] OR “occurance”[All Fields] OR “occured”[All Fields] OR “occurence”[All Fields] OR “occurences”[All Fields] OR “occuring”[All Fields] OR “occurred”[All Fields] OR “occurring”[All Fields] OR “occurs”[All Fields]))
Scopus	350	TITLE-ABS-KEY((Salmonella Infantis OR *S*. Infantis) AND (chicken OR gallus OR broiler OR bird) AND (incidence OR prevalence OR isolation OR survey OR detection OR occurence))
Web of Science	268	TS = ((Salmonella Infantis OR *S*. Infantis) AND (chicken OR gallus OR broiler OR bird) AND (incidence OR prevalence OR isolation OR survey OR detection OR occurence))

**Table 4:** Methods for the detection of antimicrobial resistance of *S.* Infantis employed in the eligible studies.

Reference	Antimicrobial Resistance Diagnostics Methods	Comment
Mughini-Gras et al. [22]	Broth microdilution	The following antimicrobials were evaluated: ampicillin, azithromycin, cefotaxime, ceftazidime, chloramphenicol, ciprofloxacin, colistin, gentamicin, meropenem, nalidixic acid, sulphamethoxazole, tetracycline, tigecycline and trimethoprim. The epidemiological cutoff values published by EUCAST were applied.
Burnett et al. [40]	Broth microdilution	The following antimicrobials were evaluated: sulfamethoxazole, trimethoprim, gentamicin, ciprofloxacin, nalidixic acid, ampicillin, cefotaxime, ceftazidime, tetracycline, chloramphenicol, colistin, azithromycin, tigecycline and meropenem. The epidemiological cutoff values (ECOFF) published by EUCAST were used to determine the presence and level of phenotypic resistance in the *Salmonella* isolates. For those antibiotics for which ECOFF values are not published (sulfamethoxazole, colistin, and azithromycin), clinical breakpoint values from the Clinical and Laboratory Standards Institute (CLSI) or previously recommended criteria were used. *Escherichia coli* ATCC 25,922 was used as the quality control strain.
El Hage et al. [44]	Antimicrobial susceptibility testing (Kirby–Bauer disk diffusion method), broth microdilution	The Kirby–Bauer disk diffusion method was used for the following antimicrobials: ampicillin, amoxicillin-clavulanic acid, piperacillin–tazobactam, cephalothin, cefuroxime, cefoxitin, cefotaxime, ceftriaxone, ceftazidime, ceftiofur, cefepime, imipenem, aztreonam, gentamycin, tobramycin, streptomycin, amikacin, netilmicin, nalidixic acid, ciprofloxacin, norfloxacin, enrofloxacin, trimethoprim, trimethoprim–sulfamethoxazole, tetracycline, and chloramphenicol. The minimum inhibitory concentrations (MICs) were further determined for the resistant isolates: cephalothin, cefuroxime, cefotaxime, ceftriaxone, ceftazidime, ceftiofur, gentamycin, nalidixic acid, ciprofloxacin, norfloxacin and enrofloxacin. *E. coli* ATCC 25,922 was used as a quality control strain.
Sasaki et al. [41]	Agar dilution method	The following antimicrobials were evaluated: ampicillin, cefazolin, ceftiofur, dihydrostreptomycin, kanamycin, oxytetracycline, bicozamycin, chloramphenicol, colistin, nalidixic acid, and trimethoprim. The resistance breakpoints defined by the CLSI were used. *E. coli* ATCC 25,922 was used as a quality control strain.
Long et al. [42]	Antimicrobial susceptibility testing (Kirby–Bauer disk diffusion method)	The following antimicrobials were evaluated: ampicillin, chloramphenicol, furazolidone, gentamicin, kanamycin, neomycin, polymyxin B, streptomycin, triple sulfa, sulfamethoxazole, trimethoprim, and tetracycline.
Badouei et al. [48]	Agar disk diffusion method	The following antimicrobials were evaluated: amoxicillin–clavulanic acid, amoxicillin, cefixime, ceftriaxone, cefazolin, chloramphenicol, chlortetracycline, ciprofloxacin, colistin, difloxacin, doxycycline, enrofloxacin, florfenicol, flumequine, fosfomycin, furazolidone, gentamicin, kanamycin, linco-spectin, nalidixic acid, neomycin, nitrofurantoin, norfloxacin, oxytetracycline, streptomycin, tetracycline and trimethoprim–sulfamethoxazole. The interpretation of results was conducted according to the Clinical and Laboratory Standards Institute (CLSI) guidelines.
Cui et al. [50]	Kirby-Bauer disk diffusion method	The following antimicrobials were evaluated: amoxicillin–clavulanic acid, nalidixic acid, ampicillin, cefazolin, doxycycline, gentamicin, trimethoprim–sulfamethoxazole, ceftazidime, chloramphenicol, ciprofloxacin, meropenem and polymyxin B. The interpretation of results was conducted according to the CLSI guidelines. *E. coli* ATCC 25,922 was used as a quality control strain.
Ishihara et al. [49]	Standardized agar dilution method, polymerase chain reaction (PCR)	The following antimicrobials were evaluated: ampicillin, ceftiofur, apramycin, dihydrostreptomycin, kanamycin, gentamicin, oxytetracycline, bicozamycin, chloramphenicol, colistin, nalidixic acid, enrofloxacin, ofloxacin, trimethoprim and sulfadimethoxine. *E. coli* NIHJ and *Staphylococcus aureus* 209P were used for quality control. MIC-resistant breakpoints were defined microbiologically when the MIC distribution of antimicrobials was bimodal. PCR was performed for *Salmonella enterica* Senftenberg, which was resistant to ceftiofur, but not for *S.* Infantis.
Khan et al. [47]	Disk diffusion method	The following antimicrobials were evaluated: amoxicillin–clavulanic acid, doxycycline, ceftriaxone, gentamicin, kanamycin, chloramphenicol, sulfamethoxazole–trimethoprim and ciprofloxacin. The interpretation of results was conducted according to the CLSI guidelines.

## Data Availability

All data produced is provided in the manuscript and in Supplementary Materials.

## Supplementary Materials

The following supporting information can be downloaded at: https://www.mdpi.com/article/10.3390/ani14233453/s1, Table S1: Screening titles and abstracts and inclusion and exclusion criteria for full text review.; Table S2: PRISMA 2020 checklist [91].
